# Polymorphisms within Genes Involved in Regulation of the NF-κB Pathway in Patients with Rheumatoid Arthritis

**DOI:** 10.3390/ijms18071432

**Published:** 2017-07-04

**Authors:** Katarzyna Gębura, Jerzy Świerkot, Barbara Wysoczańska, Lucyna Korman, Beata Nowak, Piotr Wiland, Katarzyna Bogunia-Kubik

**Affiliations:** 1 Laboratory of Clinical Immunogenetics and Pharmacogenetics, Hirszfeld Institute of Immunology and Experimental Therapy, Polish Academy of Sciences, 53-114 Wroclaw, Poland; katarzyna.gebura@iitd.pan.wroc.pl (K.G.); wysocz@iitd.pan.wroc.pl (B.W.); 2 Department of Rheumatology and Internal Medicine, Wroclaw Medical University, 50-556 Wrocław, Poland; jurekswierkot0@poczta.onet.pl (J.Ś.); lucyna_korman@wp.pl (L.K.); pwiland1@gmail.com (P.W.); 3 Department of Pharmacology, Wroclaw Medical University, 50-556 Wrocław, Poland; betanowak@interia.pl; 4 Department of Rheumatology and Internal Medicine, Wroclaw University Hospital, 50-556 Wrocław, Poland; 5 Department of Internal, Occupational Diseases, Hypertension and Clinical Oncology, Wroclaw Medical University, 50-556 Wrocław, Poland

**Keywords:** disease association, gene polymorphism, nuclear factor-κB pathway, rheumatoid arthritis, Toll-like receptors

## Abstract

Genes involved in regulation of the nuclear factor-κB (NF-κB)—pathway are suggested to play a role in pathogenesis of rheumatoid arthritis (RA). In the present study, genetic polymorphisms of *TLR2*, *TLR4*, *TLR9* and *NF-κB1* genes were investigated to assess their associations with RA susceptibility, progression and response to anti-TNF-α therapy. A group of 110 RA patients and 126 healthy individuals were genotyped for *TLR2* (rs111200466), *TLR4* (rs4986790, rs4986791), *TLR9* (rs5743836, rs187084) and *NF-κB1* (rs28362491) alleles. The presence of the *TLR9* −1486 *T* variant (*p* < 0.0001) and its homozygosity (*p* < 0.0001) were found to be associated with disease susceptibility. The *TLR9* −1237 *C* allele was associated with predisposition to RA in females only (*p* = 0.005). Moreover, the *TLR4* rs4986791 *G* (rs4986790 *T*) alleles were more frequently detected among patients with the stage IV disease (*p* = 0.045), and were associated with more effective response to anti-TNF-α therapy (*p* = 0.012). More efficient response to anti-TNF-α treatment was also observed in patients with *del* within the *NF-κB1* gene (*p* = 0.047), while for the *TLR9* −1486 *T* homozygotes, the treatment was ineffective (*p* = 0.018). *TLR* polymorphisms affect disease susceptibility and response to therapy with TNF-α inhibitors in RA patients of Caucasian origin.

## 1. Introduction

Rheumatoid arthritis (RA) is a chronic autoimmune disorder characterized by systemic inflammation and persistent synovitis that affects joints and promotes joint destruction [1]. The signal cascade mediated by Toll-Like Receptors (TLRs) and the nuclear factor κB (NF-κB) results in an immune response via rapidly increased production of cytokines, such as TNF-α, and chemokines, that may lead to inflammatory and joint destructive process.

TLRs, a family of phylogenetically conserved receptors that recognize self- and non-self-molecular patterns, which have a critical function in both innate and adaptive immune responses [2] and have been suggested to play a role in RA. High expression of TLRs 1–6 by synovial fibroblasts has been observed at an early stage of RA [3]. TLR3 and TLR4 constituted the most abundant TLRs in synovial fibroblasts and their expression levels were comparable to the levels of patients with longstanding disease. Moreover, one of the *TLR4* gene polymorphisms was reported to impair response to single disease-modifying antirheumatic drug (DMARD) treatment in recent-onset RA [4].

The nuclear factor-κB (NF-κB) family of transcription factors was identified more than 20 years ago [5]. The NF-κB is an important regulator of innate and adaptive immune responses, and it affects expression of hundreds of genes involved in regulation of proliferation, survival, stress responses, angiogenesis, inflammation and even malignant transformation. The transcription factor NF-κB is a central regulator of inflammation and can be activated by TLRs. TLR2 and TLR9 activate inflammation through the canonical NF-κB pathway, while TLR4 is involved in activation of inflammation through the canonical or non-canonical NF-κB pathway. The TLRs initiate a kinase cascade that ultimately activates the IκB kinase (IKK) complex, which phosphorylates and degrades the NF-κB inhibitor IκBa. NF-κB is shuttled from the cytosol to the nucleus, where it initiates expression of pro- and anti-inflammatory cytokines [6].

Our and other recent studies have documented that polymorphisms located within genes encoding cytokines regulated by NF-κB, IL-17A and IL-17F [7,8,9], or by TNF-α and its receptor [10], may be associated with RA susceptibility and response to therapy with TNF-α inhibitors. Clinical factors only partly explain variation in response to anti-TNF therapy. It has been suggested that gender is probably not a predictor of response, but disease activity and poor functional ability at baseline could be [11].

The present study aimed to assess the effect of the polymorphisms in *TLR2*, *TLR4*, *TLR9* and *NF-κB1* genes, involved in regulation of the NF-κB pathway, on susceptibility to RA, progression of the disease and response to therapy with TNF-α blocking agents.

For this purpose, 110 patients with high disease activity (the 28-joint Disease Activity Score; DAS 28 > 5.1) at baseline and 126 healthy individuals were investigated and typed for the *TLR2* (rs111200466, −196/−174 del/ins), *TLR4* (rs4986790, Asp299Gly, 13,843 A > G; rs4986791, Thr399Ile, 14,143 C > T), *TLR9* (rs5743836, −1237 C > T; rs187084, −1486 T > C) and *NF-κB1* (rs28362491, −94 ins/del ATTG) alleles.

## 2. Results

### 2.1. Response to Treatment

Clinical data of 87 Caucasian patients with RA treated with TNF-α inhibitors were analyzed. Among them, 50% were treated with etanercept (ETA), 36% with adalimumab (ADA), 8% with infliximab (INF) and 6% with certolizumab pegol (CER) (Table 1). Mean DAS28 at the onset of biological treatment was 6.59 ± 0.73 (range 5.14–8.05). Among subgroups treated with different anti-TNF agents, DAS28 values at the beginning were as follows: ETA—6.64 ± 0.74, ADA—6.54 ± 0.81, INF—6.64 ± 0.62, CER—6.53 ± 0.43 (ns). Mean DAS28 after 24 weeks of treatment was 4.0 ± 1.12 (range 1.97–6.88) for the whole group of patients, while in the subgroups treated with TNF inhibitors DAS28 were: ETA—3.84 ± 1.13, ADA—4.11 ± 0.95, INF—4.87 ± 1.71, CER—3.34 ± 0.14 (ns).

### 2.2. Distribution of Alleles and Genotypes of TLRs and NF-κB Encoding Genes in RA Patients and Controls, Associations with Disease Susceptibility and Progression

All allelic variants were detected in both groups of individuals studied. Minor allele frequency (MAFs) values (Table 2) were similar in patients and controls, except for the TLR9 (rs187084; −1486 T > C) polymorphism.

The TLR9 −1237 C wild type allele was more frequently detected among female patients than controls. This allele was detected in 23 out of 87 (26.44%) female patients with RA and in 5 out of 63 (7.94%) healthy females (OR = 3.816, *p* = 0.005). Thus, the *TLR9* gene (rs5743836, −1237 C > T) substitution seemed to be associated with predisposition to the disease in females only.

**Figure 1:**
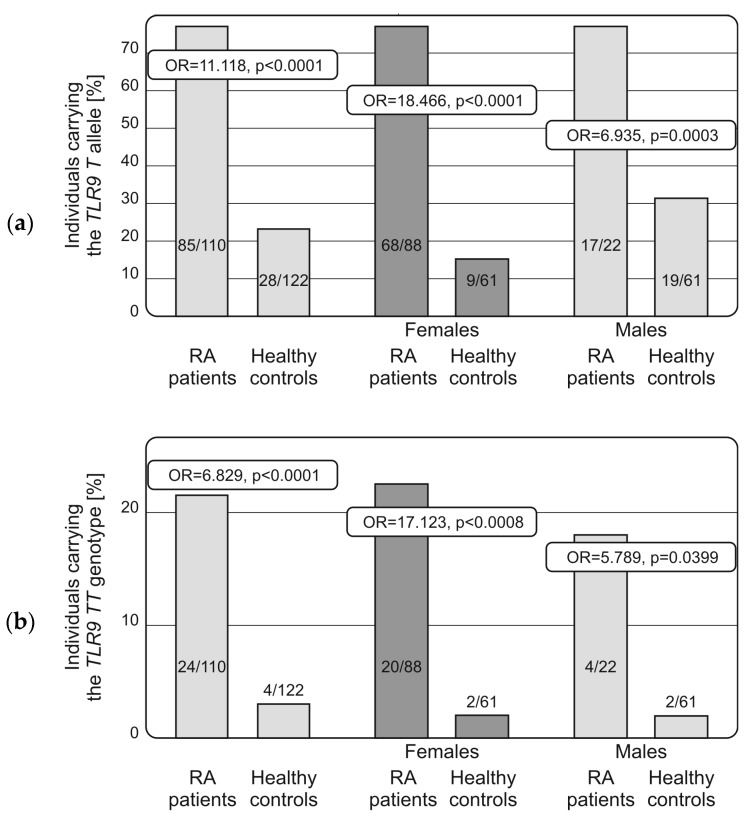
Distribution of the TLR9 (rs187084) T allele (**a**) and TT homozygous genotype (**b**) in female and male patients with rheumatoid arthritis and healthy individuals. The presence of the T allele and its homozygosity was found to be associated with predisposition to the disease in females and males.

Interestingly, the TLR9 −1486 T allele was more frequently detected among patients with RA than healthy individuals, independently of the sex (OR = 11.118, *p* < 0.001, Table 3; OR = 18.466 and OR = 6.935 for females and males, respectively, with *p* < 0.0001 in both cases, Figure 1).

Similarly, the TT homozygosity was found to be associated with RA susceptibility and detected in 24 out of 110 patients and 4 out of 112 controls (OR = 6.829, *p* < 0.001, Table 3), respectively. This relationship was independent of the sex (OR = 7.123, *p* = 0.0008 and OR = 5.789, *p* = 0.0399 for females and males, respectively, Figure 1).

There were no significant differences in the distribution of the NF-κB1 (rs28362491; −94 ins/del ATTG), TLR2 (rs201786064, −196/−174 del/ins) and TLR4 (rs4986790; Asp299Gly, 13,843 A > G; and rs4986791, Thr399Ile, 14,143 C > T) alleles and genotypes between the groups of RA patients and healthy controls (Table 2). The analyzed non-synonymous TLR4 polymorphisms were in strong linkage disequilibrium in both patients and healthy individuals. The TLR4 rs4986790 A allele was detected together with the C allele of the TLR4 rs4986791 and, in an analogous manner, the G allele—alongside the T allele. Only two individuals within the control group differed and were characterized by the rs498679 AA/rs4986791 CT haplotype.

**Figure 2:**
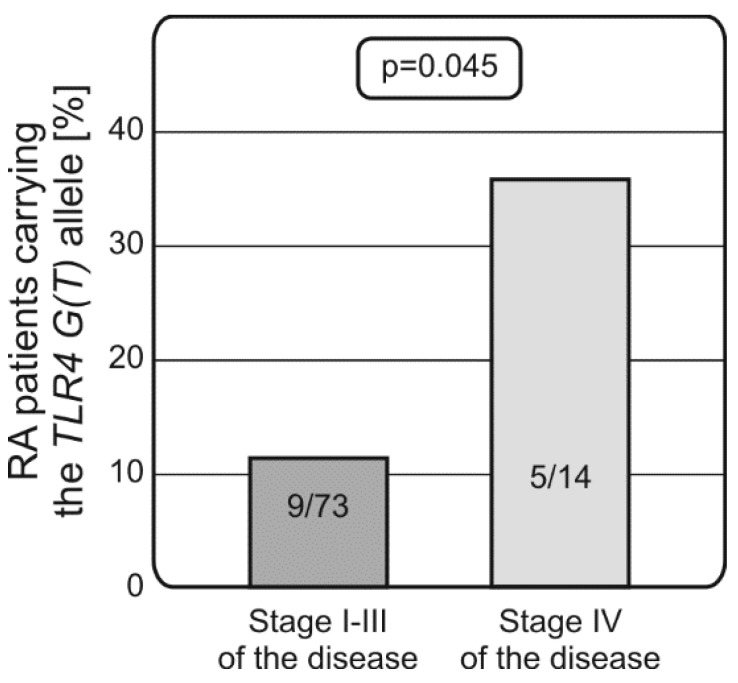
Both nonsynonymous TLR4 (rs4986790; Asp299Gly, 13,843 A > G; and rs4986791, Thr399Ile, 14,143 C > T) polymorphisms are associated with predisposition to stage IV of RA. The presence of the TLR4 rs4986791 G (rs4986790 T) allele was more frequently detected in patients presenting with stage IV of the disease.

Interestingly, patients carrying the TLR4 rs4986790 G (rs4986791 T) alleles were more likely to display stage IV of the disease (0.36 vs. 0.12, *p* = 0.045, Figure 2).

However, no significant relationship was observed between any of the polymorphisms studied and RA diagnostic markers (RF, anti-CCP, CRP).

### 2.3. Genetic Variability within the TLRs and NF-κB1 Genes and Response to Therapy with TNF-α Blocking Agents

Analyses of the response to therapy with TNF inhibitors in relation to the studied genetic polymorphisms within the TLRs and NF-κB encoding genes showed some significant associations.

Worse (EULAR) response to the anti-TNF-α therapy after 6 months of treatment was observed in patients carrying the TLR9 −1486 TT homozygous genotype (11/60 patients with DAS28 <5.1 vs. 6/11 patients with DAS28 >5.1 after 6 months of treatment, *p* = 0.018). Of note, this homozygosity was found to be associated with RA manifestation in both females and males.

Moreover, patients carrying the TLR4 rs4986790 G (rs4986791 T) alleles (more frequently present among patients in stage IV disease) were characterized by less effective response to the anti-TNF-α treatment (*p* = 0.012), similarly to those carrying the ATTG insertion in the *NF-κB1* gene (rs28362491; −94 ins/del). The ins/ins homozygosity within the *NF-κB1* gene, associated with increased promoter activity (and thus a higher expression of proinflammatory cytokines), was more frequently detected among RA patients with inefficient response to anti-TNF treatment (39% vs. 17%, *p* = 0.047). The presence of the del allele increased this efficiency and the NF-κB1 heterozygosity showed the most promising effect (*p* = 0.005).

## 3. Discussion

In the present study, we aimed to find out whether polymorphic variants of the gene coding for TLR2, TLR4 and TLR9 innate receptors, as well as NF-κB1 transcription factor, could be associated with predisposition to rheumatoid arthritis, progression of the disease, and response to anti-TNF treatment. The previous studies have suggested an influence of these polymorphisms on the innate immune response and etiology of some autoimmune diseases, however, none of those studies concerned Polish populations.

Some potential associations of polymorphic variants of the genes coding for TLRs with RA susceptibility in various populations have been described [12,13,14]. A relationship between *TLR*3 rs3775291 genetic variants and sero-negative RA has been reported in Caucasian population from Denmark [12]. Also, a significant correlation of SNPs rs2072493 in *TLR5* and rs3853839 in *TLR7* with RA risk was observed in Dutch population, however replication cohorts of this study did not confirm these findings [13].

The *TLR4* polymorphism resulting in the Asp299Gly amino acid substitution (rs4986790) did not appear to affect susceptibility to RA in UK patients [15], nor in 5 other European studies [16], which also concurs with our current observations. Additionally, a recently published meta-analysis did not show any significant association of this SNP with predisposition to RA [14].

In the current study, the presence of the *TLR4* rs4986790 *G* (rs4986791 *T*) allele, resulting in 299Gly (399Ile), was more frequently detected among patients with stage IV disease, and was also associated with more effective response to anti-TNF-α therapy. In line with our observations, Kuuliala et al. [4] found that RA patients carrying the *TLR4 G* allele (299Gly) were characterized by significantly higher DAS28 and lower remission rate after single DMARD treatment.

Davis et al. [17] demonstrated other SNPs within the *TLR4* gene-rs1927911 to affect RA progression. In their study, disease activity measures progressed less over time in the homozygous minor allele group rs1927911. In addition, Davis et al. [17] reported an influence of *TLR4* rs1927911 polymorphism on disease progression in RA. Clinical measures of disease progression, including DAS28, correlated inversely with presence of the *TLR4* rs1927911 *TT* genotype among patients.

The results of the present study also suggest that two SNPs located within the *TLR9* gene might be involved in RA pathogenesis, supporting the hypothesis that microbial infection may play a role in RA and that the infection triggers inflammatory responses recognized by pattern recognition receptors (including TLRs). In our study, both *TLR9* polymorphisms were found to be associated with predisposition to the disease (the *TLR9* −1237 *C* allele and the *TLR9* −1486 *T* allele and its homozygosity). These relationships have not been previously described in the literature, although these SNPs were studied in context of other autoimmune disorders.

The *T* allele of *TLR9* gene polymorphism (rs5743836, −1237 C > T) was found to act as a risk factor of systemic lupus erythematosus (SLE) in South Indian Tamils [18]. The inconsistent results of this report and the present study stem from ethnic and sex from differences between studied populations.

The *TLR9* −1486 T > C polymorphism (rs187084) has also been described to be significantly associated with increased susceptibility to ophthalmopathy in Taiwanese male patients with Graves’ disease [19]. In addition, relationships between two other *TLR9* gene polymorphisms (rs352139, rs352140) and lupus nephritis development in a Chinese Han population were observed [20].

Interestingly, Bharti et al. [21] have recently found that the presence of the *TLR9* −1486 *C* allele increases transcriptional activity of the *TLR9* gene, which in turn induces high levels of Interferon gamma-induced protein 10 (IP-10) in patients with pulmonary tuberculosis; however, expression of proinflammatory cytokines such as IFNγ and TNFα was significantly lower in *TLR9 C* allele carriers as compared to those with the *TLR9* −1486 *T* allele. These results suggest that individuals carrying the *TLR9* −1486 *T* genetic variant (including the *TLR9* −1486 *TT* homozygotes) produce more IFNγ and TNFα, whose increased levels have been observed in RA patients. Moreover, our preliminary results suggested a strong tendency between *TLR9* −1486 C > T SNP and TNFα levels was observed in our study (187.787 vs. 119.608 pg/mL; *T* = 1.725, *p* = 0.0973; T-Test for 2 Independent Means; for *TT* vs. *C* carriers 3 months after anti-TNF treatment, respectively). Of note, both *TLR9* −1486 *T* allele and its homozygosity correlated with predisposition to RA development while the *TLR9* −1486 *T* homozygous genotype was associated with less favorable response to therapy with TNFα inhibitors.

According to our current knowledge, there has been only one published study to date suggesting the potential role of the *TLRs* genes in response to anti-TNFα treatment. Coenen et al. [13] reported the association of the SNP rs2072493 in *TLR5* gene with anti-TNF treatment outcome in Dutch patients with RA however this relationship wasn’t observed in the Swedish population. There have been also no previous studies showing the effect of the *NF-κB1* genetic variants on the anti-TNF therapy outcome, although our results suggest that the presence of the *del* allele within the *NF-κB1* gene favors positive response to therapy with TNFα blocking agents.

Taken together, the present study contributes to the reports on genetic factors associated with RA and broaden our knowledge regarding RA pathogenesis. Our results suggest that 2 out of 6 polymorphisms studied, SNPs rs5743836 and rs187084 within the *TLR9* gene, could be considered valuable prediction factors in estimation of the risk for RA development and prognosis. Moreover, anti-TNFα treatment outcome was found to be affected by both *TLR9* SNPs (rs5743836 and rs187084), *TLR4* non-synonymous substitutions and ins/del polymorphisms within the *NF-κB1* gene.

Obviously, due to relatively small number of individuals investigated, our findings need to be confirmed by extended studies involving an independent cohort of patients before drawing the final conclusions. It would be also interesting to relate polymorphisms and expression/protein production of the investigated genes in RA patients with different manifestations of the disease and response to therapy with TNFα blocking agents. Further studies are warranted to elucidate the role of the *TLR9* and *TLR2* polymorphisms in pathogenesis of RA.

## 4. Materials and Methods

### 4.1. Patients and Controls

One hundred and ten patients (female/male: 87/23) diagnosed with RA were investigated. The following inclusion criteria were used: consent to participate in the study; confirmed RA based on the 2010 American College of Rheumatology/European League Against Rheumatism (EULAR) classification criteria; active form of the disease: DAS 28 > 5.1; age over 18; women and men with reproductive potential had to use reliable contraception; taking NSAIDs and glucocorticosteroids in stable doses was allowed.

The following exclusion criteria were used: pregnancy or breastfeeding; coexistence of other systemic diseases of connective tissue besides RA; clinically significant impairment of hepatic or renal function; alcohol abuse; infection with hepatotropic viruses; infections resistant to therapy; ongoing history of cancer if no cure was achieved; uncontrolled diabetes; patient unwilling or unable to cooperate.

Patients who had been treated with recommended doses of TNF-α inhibitors (adalimumab, etanercept, infliximab, certolizumab) for at least 3 months or had stopped therapy because of adverse events were investigated. To examine the response to anti-TNF therapy in RA, blood samples, laboratory data and clinical data were collected at baseline (prior to anti-TNF therapy), 3 and 6 months after treatment. Clinical evaluation was based on medical history, number of painful and swollen joints, pain intensity assessed by the patient on a 100 mm visual analogue scale (VAS) and laboratory tests (ESR, CRP). The parameters made it possible to determine improvement according to the criteria based on DAS 28, suggested by the EULAR.

One hundred and ten patients were analyzed for disease susceptibility (in comparison to healthy individuals). Eighty-seven cases were investigated for associations with disease progression and response to therapy with TNF-α inhibitors (Table 1). All patients provided written informed consent. The study was approved by the Wroclaw Medical University Ethics Committee (No. KB – 625/2016, 29 December 2016) and the procedures were in accordance with the ethical standards of the Helsinki Declaration, as revised in 2013.

Stages of RA were assessed according to Wheeless [22]. Following this classification, first stage RA is characterized by synovitis or an inflammation of the synovial membrane causing swelling of involved joints and pain upon motion. However, there is no x-ray evidence of joint destruction, with the exception of swelling of soft tissues or early stages of osteoporosis. In stage II, there is a spread of inflammation in synovial tissue affecting joint cavity space across joint cartilage. This inflammation leads to gradual destruction of cartilage accompanied by narrowing of the joint. Severe RA, stage III, is marked by formation of pannus in the synovium. Loss of joint cartilage exposes bone beneath the cartilage. These changes become evident in x-rays, together with erosions and signs of deformation. Stage IV is called terminal or end stage RA. The inflammatory process has subsided and formation of fibrous tissue and/or fusing of bone results in ceased joint function. Rheumatoid nodules may also be present in patients in stage IV of the disease.

In addition, 126 healthy individuals of both sexes (female/male: 63/63) served as controls.

### 4.2. DNA Isolation

DNA was extracted from samples of peripheral blood taken on EDTA using Maxwell 16 Blood DNA Purification Kit (Promega Corp., Madison, WI, USA) or silica membranes (Qiagen, Hilden, Germany) following the recommendations of the manufacturers.

### 4.3. NF-κB1 Genotyping

The *NF-κB1* (rs28362491; −94 ins/del ATTG) polymorphism was analyzed using polymerase chain reaction combined with capillary gel electrophoresis performed in the 3500 Genetic Analyzer (Applied Biosystems, Foster City, CA, USA) instrument. Electrophoresis demonstrated the presence of a 154 bp fragment for the *del* allele and/or of a 158 bp fragment in length for the *ins* allele.

### 4.4. TLR4 Genotyping

Two SNPs within the exon 3 of the *TLR4* gene (rs4986790, Asp299Gly, 13,843 A > G; and rs4986791, Thr399Ile, 14,143 C > T) were determined by real-time polymerase chain reaction (PCR) amplifications using LightSNiP assays designed for both polymorphisms by TIB MOLBIOL (Berlin, Germany). The reaction was performed following the recommendation of the manufacturer on the LightCycler 480 instrument (Roche Diagnostics GmbH, Basel, Switzerland).

### 4.5. TLR2 Genotyping

The PCR was employed for analysis of the 23 bp del/ins polymorphism within the *TLR2* (rs111200466, −196/−174 del/ins) gene using the following oligonucleotides: forward primer: 5′-GCG GAC TTT CCC TTT TGC T-3′ and reverse primer: 5′-GTG CAG AGA GAC CAC ACG AG-3′. The PCR conditions were as follows: 95 °C for 5 min; 35 cycles of 95 °C for 30 s, 57 °C for 30 s and 72 °C for 30 s; and a final elongation step at 72 °C for 10 min. Amplified products of either 134 bp (deletion) or 157 bp in length (insertion) were then analyzed by electrophoresis in a 2.5% agarose gel stained with Simply Safe (EURx, Gdańsk, Poland) and visualized under UV light.

### 4.6. TLR9 Genotyping

For analysis of the rs5743836 (−1237 C > T) and rs187084 (−1486 T > C) polymorphisms in the *TLR9* gene, polymerase chain reaction restriction fragment length polymorphism (PCR-RFLP) assay was employed [23]. Sequences of forward primers for the *TLR9* rs5743836 and rs187084 genotypings were 5′-ATG GGA GCA GAG ACA TAA TGG A-3′ and 5′-TCC CAG CAG CAA CAA TTC ATT A-3′, respectively. The common reverse primer 5′-CTG CTT GCA GTT GAC TGT GT-3′ was used for both analyses. The PCR conditions were alike for both rs5743836 and rs187084 polymorphisms: 95 °C for 5 min; 35 cycles of 95 °C for 30 s, 60 °C for 30 s and 72 °C for 30 s; and finally, 72 °C for 10 min. Validations of the amplification products of 135 bp in length for the rs5743836 and 499 bp for the rs187084 polymorphism were performed via electrophoresis in a 2% agarose gel with Simply Safe (EURx, Gdańsk, Poland) and visualization in the UV light. The next step was digestion of the PCR products with either *BstN*I (for the rs5743836 polymorphism) or *Afl*II restriction endonuclease (for the rs187084 polymorphism) (both from New England BioLabs Inc., Ipswich, MA, USA). Analysis of the final products was performed in a 3% agarose gel stained with Simply Safe (EURx, Poland) and visualized under UV light. The observed patterns were 60 bp, 48 bp and 27 bp for the *C* allele or 108 bp and 27 bp for the *T* allele of the *TLR9* rs5743836 polymorphism. As for the rs187084 polymorphism in the *TLR9* gene, final products were of 499 bp for the *C* allele or 172 bp and 327 bp in length for the *T* allele.

### 4.7. Statistical Analysis

Genotype and allele frequencies were compared between the study groups by the Chi^2^ test with Yates correction or Fisher’s exact test when necessary, using online tools (Available online: http://www.socscistatistics.com/tests/Default.aspx). The odds ratio (OR) was calculated by Haldane’s modification of Woolf’s method and the significance of its deviation from unity was estimated by Fisher’s exact test. Probability values <0.01 were considered statistically significant, and those between 0.05 and 0.1 as indicative of a trend.

## Figures and Tables

**Table 1:** Characteristics of RA patients for whom associations of single nucleotide polymorphisms with disease progression and response to therapy with TNF-α inhibitors were analyzed.

RA Patients (*N* =)	87
Sex (female/male); *n* (%)	71 (82%)/16 (18%)
Age (years)	50.7 ± 12.3 (range: 17–77)
Females (%)	71 (82%)
Disease duration (years)	12.4 ± 8.3 (range 1–39)
Disease onset (years)	38.8 ± 12.0 (range 15–65)
Current smokers (%)	14
RF+ Rheumatoid factor positive, *n* (%)	72
ACPA+/Anti-CCP present, *n* (%)	47
Stage, *n* (%)	
1	2 (2.3%)
2	20 (23%)
3	51 (58.6%)
4	14 (16.1%)
DAS28 at baseline	6.59 ± 0.73 (range 5.14–8.05)
DAS28 at week 24	4.0 ± 1.12 (range 1.97–6.88)
anti-TNF drug	
etanercept (%)	44 (50%)
adalimumab (%)	32 (36%)
infliximab (%)	7 (8%)
certolizumab pegol (%)	5 (6%)
Glucocorticosteroids %	79 (mean dose 9.3 mg prednisone daily)
Methotrexate %	71 (mean dose 20.4 mg weekly)

RA—rheumatoid arthritis, RF—rheumatoid factor, ACPA—anti-citrullinated protein antibodies, DAS 28—disease activity score 28, TNF—tumour necrosis factor.

**Table 2:** Minor allele frequencies (MAFs) of the polymorphisms studied in Polish patients with rheumatoid arthritis and healthy individuals.

Polymorphism	Minor Allele	MAF RA Patients	MAF Controls
*NF-κB1* (rs28362491, −94 del/ins ATTG)	*del*	0.423	0.385
*TLR2* (rs111200466, −196/−174 del/ins)	*del*	0.168	0.179
*TLR4* (rs4986790, 13843 A > G)	*G*	0.077	0.065
*TLR4* (rs4986791, 14143 C > T)	*T*	0.077	0.074
*TLR9* (rs5743836; −1237 C > T)	*C*	0.135	0.083
*TLR9* (rs187084; −1486 T > C)	*T*	0.495	0.131

Of the six polymorphisms studied, 2 single nucleotide polymorphisms (SNP) within the *TLR9* gene (rs5743836, −1237 C > T and rs187084, −1486 T > C) were found to be associated with predisposition to the disease. Distribution of alleles and genotypes of the *TLR2*, *TLR4*, *TLR9* and *NF-κB1* genes is given in Table 3.

**Table 3:** Distribution of the TLRs and NF-κB1 alleles and genotypes in Polish patients with rheumatoid arthritis and healthy individuals. The TLR2 and TLR9 polymorphisms were associated with predisposition to RA. HWE stands for Chi^2^ *p*-values calculated for Hardy-Weinberg Equilibrium assessment.

Polymorphism	RA Patients		Controls		OR, *p*
	*n*	(%)	*n*	(%)	
***NF-κB1* (rs28362491, −94 del/ins ATTG)**					
*del/del*	19	17.3	14	11.1	
*del/ins*	55	50.0	69	54.8	
*ins/ins*	36	32.7	43	34.1	
*del*	74	67.3	83	65.9	
*ins*	91	82.7	112	88.9	
HWE	*p* = 0.798		*p* = 0.079		
***TLR2* (rs111200466, −196/−174 del/ins)**					
*del/del*	1	1.8	7	5.5	
*del/ins*	35	31.8	31	24.6	
*ins/ins*	74	67.3	88	69.8	
*del*	36	32.7	39	30.9	
*ins*	109	90.1	119	94.4	
HWE	*p* = 0.150		*p* = 0.070		
***TLR4* (rs4986790, Asp299Gly, 13843 A > G)**					
*AA*	94	85.5	107	87.0	
*AG*	15	13.6	16	13.0	
*GG*	1	0.9	0	0.0	
*A*	109	99.1	123	100.0	
*G*	16	14.5	16	13.0	
HWE	*p* = 0.646		*p* = 0.440		
***TLR4* (rs4986791, Thr399Ile, 14,143 C > T)**					
*CC*	94	85.5	104	85.2	
*CT*	15	13.6	18	14.8	
*TT*	1	0.9	0	0.0	
*C*	109	99.1	122	100.0	
*T*	16	14.5	0	0.0	
HWE	*p* = 0.646		*p* = 0.379		
***TLR9* (rs5743836; −1237 C > T)**					
*CC*	1	0.9	0	0.0	
*CT*	28	25.2	21	16.7	
*TT*	81	73.9	105	83.3	
*C*	29	26.4	21	16.7	(association in females only) ^#^
*T*	109	99.1	105	83.3	
HWE	*p* = 0.397		*p* = 0.308		
***TLR9* (rs187084; −1486 T > C)**					
*TT*	24	21.8	4	3.3	OR = 6.829, *p* < 0.0001
*TC*	61	55.5	24	19.7	OR = 4.995, *p* < 0.0001
*CC*	25	22.7	94	77.0	
*T*	85	77.3	28	22.9	OR = 11.118, *p* < 0.0001
*C*	86	78.2	118	96.7	
HWE	*p* = 0.252		*p* = 0.131		

^#^ Association of the C allele with RA risk: OR = 3.816, *p* = 0.005 observed in females only (23 out of 87 (26.44%) female patients with RA vs. 5 out 63 (7.94%) of healthy females).

## Abbreviations

DAS 28	28-Joint Disease Activity Score
DMARD	Disease-Modifying Antirheumatic Drug
EULAR	European League Against Rheumatism
HWE	Hardy-Weinberg Equilibrium
IKK	IκB Kinase
MAF	Minor Allele Frequency
NF-κB	Nuclear Factor-κB
OR	Odds Ratio
*p*	Probability
RA	Rheumatoid Arthritis
RF	Rheumatoid Factor
SNP	Single Nucleotide Polymorphism
TLR	Toll-Like Receptor
TNF-α	Tumor Necrosis Factor α
VAS	Visual Analogue Scale
